# Establishment and assessment of an amplicon sequencing method targeting the 16S-ITS-23S rRNA operon for analysis of the equine gut microbiome

**DOI:** 10.1038/s41598-021-91425-7

**Published:** 2021-06-04

**Authors:** Yuta Kinoshita, Hidekazu Niwa, Eri Uchida-Fujii, Toshio Nukada

**Affiliations:** Microbiology Division, Equine Research Institute, Japan Racing Association, 1400-4 Shiba, Shimotsuke, Tochigi 329-0412 Japan

**Keywords:** Metagenomics, Bacteriology, Metagenomics, Metagenomics

## Abstract

Microbial communities are commonly studied by using amplicon sequencing of part of the 16S rRNA gene. Sequencing of the full-length 16S rRNA gene can provide higher taxonomic resolution and accuracy. To obtain even higher taxonomic resolution, with as few false-positives as possible, we assessed a method using long amplicon sequencing targeting the rRNA operon combined with a CCMetagen pipeline. Taxonomic assignment had > 90% accuracy at the species level in a mock sample and at the family level in equine fecal samples, generating similar taxonomic composition as shotgun sequencing. The rRNA operon amplicon sequencing of equine fecal samples underestimated compositional percentages of bacterial strains containing unlinked rRNA genes by a fourth to a third, but unlinked rRNA genes had a limited effect on the overall results. The rRNA operon amplicon sequencing with the A519F + U2428R primer set was able to detect some kind of archaeal genomes such as *Methanobacteriales* and *Methanomicrobiales*, whereas full-length 16S rRNA with 27F + 1492R could not. Therefore, we conclude that amplicon sequencing targeting the rRNA operon captures more detailed variations of equine microbiota.

## Introduction

The horse is a hindgut fermenter, and most microbial activities in the gastrointestinal (GI) tract take place in the large intestine, so the majority of intestinal microbes reside in the colon and the comparatively enlarged caecum^1^. The degradation of indigestible cellulosic and hemi-cellulosic forage components by these microbes is crucial for the bioavailability of energy and essential nutrients in horses^2^. Studies of these activities in the GI tract of horses have focused on bacteria, but fungi and archaea are also involved^3,4^. In particular, methanogenic archaea, widely found in the equine hindgut, metabolize H_2_ and CO_2_ to methane, and are considered to support the carbohydrate-degrading activity of cellulolytic bacteria in the hindgut^4–6^. The GI microbiota also plays a crucial role in maintaining normal microbial flora by various methods: it produces antimicrobial products, competes directly for nutrients with pathogens, inhibits or inactivates bacterial toxins, and produces bacteriocins and short-chain fatty acids, which inhibit the growth of pathogens and pathobionts^7^. It has also been shown to modify virulence factor expression of pathogens and to facilitate the host barrier function through the upregulation of mucus production, antimicrobial molecules, and secretion of IgA^7^.

High-throughput sequencing technologies are widely used to investigate microbial flora in many environments, such as oral, intestinal, vaginal, aquatic, and soil. Amplicon sequencing targeting a specific region in microbial genomes is one methodology that uses high-throughput sequencing, and a partial region of the 16S rRNA gene is commonly used to study microbiomes^8^. The third generation of sequencing technologies now allows the production of very long reads, albeit with higher error rates, in real time^9,10^. Yet despite the higher error rates, analysis of the full-length 16S rRNA gene amplicon by nanopore sequencing technology has a comparable or higher taxonomic resolution of bacterial flora at the genus and particularly species levels than short-read sequencing on an Illumina platform^11–13^. Long amplicon analysis targeting the nearly complete 16S-ITS-23S rRNA operon has been used to achieve greater confidence in taxonomic assignment^14–16^, but it could miss some proportion of bacterial species on account of unlinked rRNA genes. Unlinked rRNA genes are widespread among bacteria and archaea: at rates of 13–41% in soil, 7.7–29% in sediment, and 8.1–8.8% in anaerobic digesters, though not in the human gut^17^. In addition, the incompleteness of databases for the 16S-ITS-23S rRNA operon could be a limitation in using the operon region in metagenome sequencing; i.e., some false-positives could be assigned owing to an insufficient record of bacterial and archaeal species or the short sequence lengths of the deposited microbes^14,16^.

Our main aim in this study was to reveal the features and limitations of rRNA operon amplicon sequencing in metagenomic analysis of equine fecal samples in comparison with full-length 16S rRNA gene sequencing and shotgun sequencing. First, we evaluated the two nanopore-based long amplicon sequencing methods with different PCR polymerase kits and reference databases for taxonomic assignments, using a mock bacterial community. Short-read sequence data from the Illumina MiSeq system was also evaluated in comparison to the long amplicon sequencings. Second, we explored unlinked rRNA genes in equine feces and assessed their influence on overall taxonomic compositional results generated by the rRNA operon amplicon sequencing. Finally, we compared the results to reveal the advantages and disadvantages of each sequencing method.

## Materials and methods

### Genomic DNA of mock bacterial community

A bacterial DNA cocktail of a mock community (DNA-Mock-001, Lot Number BK1903D01) was obtained from the National Institute of Technology and Evaluation’s Biological Resource Center (NBRC, Tokyo, Japan). It comprises genomic DNA prepared from 15 bacterial strains (Table S1).

### Fecal sampling and DNA extraction

Fecal samples were collected from three female Thoroughbred horses (*Equus ferus caballus*): A and S (2 years old) and L (9 years old). All horses were clinically healthy, with no apparent history of intestinal problems. No antimicrobials had been administered to them for at least a month before sampling. The samples from horses A and S were immediately sampled just after being dropped on the straw bed in the stable. The sample from horse L was collected directly from the rectum. The samples were immediately placed on ice and the genomic DNA of each was extracted within 1 h after collection. For DNA extraction, 3 g of wet fecal sample was suspended vigorously in 40 mL of PBS and then left for 1 min at room temperature to remove large plant debris. Then 4 mL of the suspension was centrifuged at 13,000×*g* for 2 min at 4 °C, and the pellet was washed twice with TE buffer containing 10 mM Tris and 1 mM EDTA. The final pellet was suspended in 800 µL of distilled water. The genomic DNA was extracted from the suspension by using a Quick-DNA Fecal/Soil Microbe Kit (Zymo Research, Tokyo, Japan) following the manufacturer’s instructions. It was purified with an Agencourt AMPure XP purifier (Beckman Coulter, Brea, CA, USA), and short DNA (< 10 kb) was depleted by using a short-read eliminator (XS; Circulomics, Baltimore, MD, USA).

All samplings and experiments were conducted in accordance with ethical and welfare regulations of the Animal care committee of the Equine Research Institute. The Animal care committee of the Equine Research Institute approved all experimental protocols. We also complied with the ARRIVE guidelines (https://arriveguidelines.org/).

### Sequencing and base-calling for mock community

For nanopore sequencing, genomic libraries for two target regions (full-length 16S rRNA gene and 16S-ITS-23S rRNA operon) were prepared by using the four-primer PCR method protocol, version FFP_9038_v108_revN_14Aug2019 (Oxford Nanopore Technologies [ONT], Oxford, UK) with slight modifications. The four-primer PCR uses two target-specific inner primers with a 5′ tail and two universal outer primers which prime off the tail on the 5′ end of the inner primers (Table S2), resulting in the generation of target amplicons with barcodes^15,18,19^. PCR amplifications were conducted using either LongAmp™ Taq 2 × Master Mix (New England Biolabs, Ipswich, MA, USA) or the KAPA2G™ Robust HotStart Ready Mix PCR Kit (Kapa Biosystems, Wilmington, MA, USA). PCR was performed in a total volume of 25 µL containing the inner primers (50 nM each), the barcoded outer primer mixture (300 nM) from the PCR barcoding kit (SQK-PBK004; ONT), and the DNA cocktail (1 ng) as template. PCR conditions are shown in Table S3. The PCR amplicons were purified with the Agencourt AMPure XP purifier and quantified by a NanoDrop spectrophotometer (ThermoFisher Scientific), and the libraries were sequenced on a MinION sequencer using R9.4.1 flow cells (FLO-MIN106D; ONT) following the manufacturer’s instructions. Base-calling of raw fast5 data from the MinION was carried out in Guppy v. 3.6.1 software (ONT) with its “–trim_barcodes” option for removing sequencing adapters and barcodes. Details on each sample, including number of reads, median read lengths, and median read quality, are shown in Table S4.

The same genomic DNA was also sequenced on the Illumina MiSeq system targeting hypervariable region (V3-V4) by a commercial service (Oral Microbiome Center, Kagawa, Japan). This sequencing was conducted with a primer set of 341F and 806R following an Illumina protocol (16S Metagenomics Sequencing Library Preparation).

### Sequencing and base-calling for fecal samples

For fecal samples, both long amplicon sequencing and shotgun sequencing were conducted. Genomic libraries for amplicon sequencing targeting the full-length 16S rRNA gene or the 16S-ITS-23S rRNA operon were prepared as for the mock community by using a KAPA2G Robust HotStart Ready Mix PCR Kit. Genomic libraries for shotgun sequencing were prepared by using a Rapid Barcoding Kit (SQK-RBK004; ONT) following the manufacturer’s instructions. All libraries were sequenced on the MinION sequencer using R9.4.1 flow cells. Base-calling of raw fast5 data was carried out as above; details are shown in Table S4.

### Sequencing data processing for mock community

The raw FASTQ files from nanopore sequencings were pretrimmed in Seqkit v. 0.12.0 software^20^ to filter by quality scores of 10, with lengths of 1300 bp for the full-length 16S rRNA gene and 3500 bp for the 16S-ITS-23S rRNA operon. Afterward, 30,000 reads were subsampled in Seqtk v. 1.3. software (https://github.com/lh3/seqtk), and chimera reads were removed in yacrd v. 0.6.1 software^21^. Twenty thousand quality-controlled reads were selected by quality score > 12 and by size: 1300–1950 bp for the full-length 16S rRNA gene and 3500–5000 bp for the 16S-ITS-23S rRNA operon. Accurate taxonomic assignments of the quality-controlled read sets were performed with the CCMetagen pipeline by coupling with KMA v. 1.3.0 and CCMetagen v. 1.2.2 software^22,23^, i.e., read mapping to a reference database in KMA software (-mem_mode, -bcNano, and -1t1 options), specifying the minimum phred score (-mp 20), minimum alignment score (-mrs 0.0), and base-calling option (-bc 0.7), followed by a quality-filtering step in CCMetagen software with default settings. Two reference databases—rrn DB^24^ and ncbi_202006 DB, described below—were used in the taxonomic assignment step. The dissimilarity indices between the percentage of each sequencing condition and that of the expected abundance of the mock community were calculated by the vegan package in R v. 3.6.1 software with default settings^25,26^.

For analysis of short-read sequencing data, paired-end reads were merged in bbmerge software embedded in bbmerge v. 38.18 with default settings^27^. The merged reads were filtered in trimmomatic v. 0.39 software^28^, specifying the following parameters: headcrop of 15 bp, tailcrop of 5 bp, window length of 4 bp, window quality of 20, and minimum length of 350 bp. Chimera reads were removed in Vsearch v. 2.17.0 software with default settings^29^ and 20,000 reads were subsampled in the Seqtk software. Taxonomic assignments of the quality-controlled read set were performed with the CCMetagen pipeline by using the ncbi_202006 DB, specifying -1t1 option in the KMA software.

### Sequencing data processing for fecal samples

For the FASTQ files from the long amplicon sequencing, pretrimmed reads were created in Seqkit software to filter by quality scores of 10, with lengths of 1300 bp for the full-length 16S rRNA gene and 3500 bp for the 16S-ITS-23S rRNA operon. Chimera reads were removed in yacrd software. Quality-controlled reads were selected by quality score > 11 and by size: 1300–1950 bp for the full-length 16S rRNA gene and 3500–5000 bp for the 16S-ITS-23S rRNA operon. Taxonomic assignments were performed as above.

For the FASTQ files from the shotgun sequencing, quality-controlled data sets were created in Seqkit software to filter by lengths of 1000 bp and quality scores of 10. After conversion from FASTQ format to FASTA format, rRNA genes were detected in each sequence read in Barrnap v. 0.9 software (https://github.com/tseemann/barrnap) with default settings, and the reads which contained rRNA genes were extracted from the raw FASTA files in Seqkit software: 2333 sequence reads from horse A, 4417 reads from horse S, and 8700 reads from horse L. The taxon assignments of each read against ncbi_202006 DB were performed as above. For further investigations of unlinked rRNA genes, the reads that could be classified to at least phylum level were kept, and we classified reads as containing unlinked rRNA genes following the previous criteria^17^, namely if there was > 1500 bp between the 16S and 23S rRNA genes, or if there was no 23S domain found by 1500 bp after the end of the 16S rRNA. We removed the sequence reads for final analyses that could not be judged to contain linked or unlinked rRNA genes (i.e., with < 1500 bp after the 3′ end of the 16S rRNA gene), and we kept only reads that included a 16S rRNA gene to avoid potential double-counting organisms with unlinked 16S and 23S rRNA genes. Information on all long-read sequences included is shown in Table S5.

The ggplot2 package in R v. 3.6.1 software was used to depict the percentages of quality-filtered reads from the CCMetagen pipeline in each taxon and boxplots for the top 20 orders with the highest average percentage^30^. The NMDS analysis was carried out using the vegan package in R software with default settings^25^. A heat map showing the percentage of each order identified was plotted in ComplexHeatmap software in R, specifying the distance measure “spearman” and clustering method “ward.D2” for hierarchical clustering^31^.

### Taxonomic reference databases

We used two databases which contain sequences of the 16S-ITS-23S rRNA operon: (1) rrn DB, which includes sequences from the whole ribosomal operon of 22,351 bacterial species retrieved from GenBank^24^, and (2) ncbi_202006 DB, which was created in this study. To create ncbi_202006 DB, all of the bacterial and archaeal data were downloaded from reference sequence database at NCBI in June 2020 in genome_updater v. 0.2.2 software (https://github.com/pirovc/genome_updater), and rRNA genes were predicted and extracted in Barrnap software. Each rRNA gene in the same nucleotide sequence was concatenated if the distance between rRNA genes was < 2000 bp, because the lengths of ITS regions in linked rRNA genes were by definition < 1500 bp^17^. All sequences whose lengths were < 1000 bp were removed from the database. The final ncbi_20206 DB consists of 493,329 sequences, including > 2700 genera and > 11,500 species of bacteria or archaea. To account for the diversity of rRNA operon sequences in the same bacterial species, multiple operon sequences of the same bacterial species are included in the ncbi_202006 DB. Accessions for ncbi_20206 DB are available at https://bitbucket.org/ykinoshita1984/ncbi_db/downloads/.

To check the impact of an inadequate database on the mapping results, we created an incomplete database by removing two bacterial species (*Bacillus subtilis* and *Lactobacillus delbrueckii*) from the ncbi_202006 DB.

### Statistical analysis

The vegan package in R software was used to conduct permutational multivariate analysis of variance (PERMANOVA), specifying the Bray–Curtis method and 10,000 permutations^25^. *P* < 0.05 was considered to indicate a significant difference.

## Results and discussion

### Mock community

**Figure 1:**
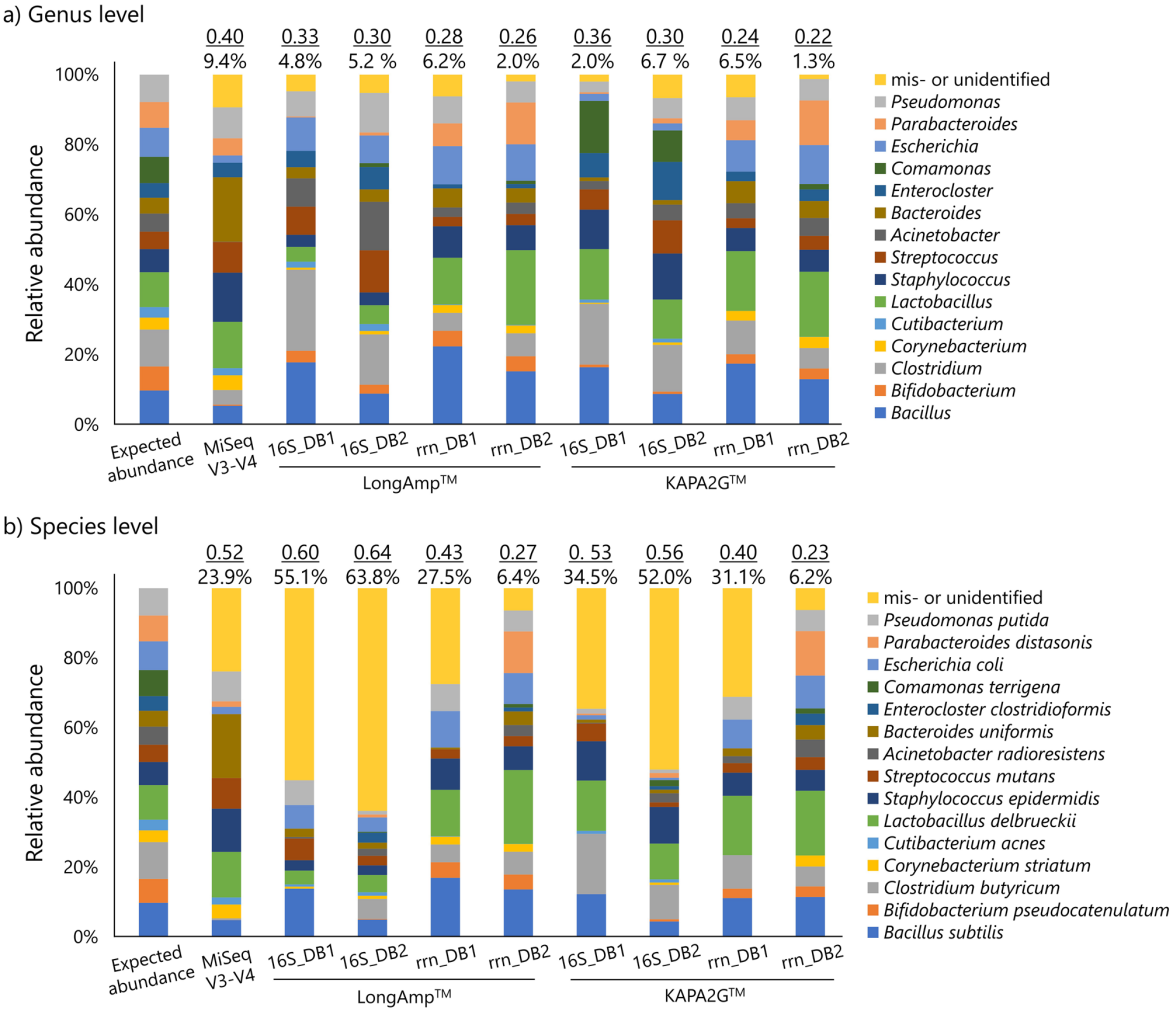
Relative abundance of mock community at (**a**) genus and (**b**) species levels. Indices of dissimilarities between the expected abundance and each sequencing condition (underlined) and the percentages of mis- or unidentified sequences are shown above each bar graph. *16S* full-length 16S rRNA gene amplicon sequencing, *rrn* rRNA operon amplicon sequencing. DB1: rrn DB^24^. DB2: ncbi_202006 DB.

Comparisons of metagenomic sequence data acquired with several combinations of conditions from defined mock communities are important for the assessment of sequencing platform performance and downstream analyses, including taxonomic assignment. Using a commercial mock community containing DNAs of 15 bacteria, we compared long-read metagenomic sequences from three sources: (1) PCR polymerase kits—LongAmp™ Taq 2 × Master Mix and KAPA2G™ Robust HotStart Ready Mix PCR Kit; (2) PCR target regions—full-length 16S rRNA gene (~ 1450 bp) and 16S-ITS-23S rRNA operon (~ 4000 bp); and (3) reference databases—the *rrn* operon database, rrn DB^24^, and the ncbi_202006 DB, newly designed in this study. The accuracy of short-read metagenomic sequence data targeting the V3-V4 region was also compared with those of the long-read sequence data. The degree of assignment of 20,000 quality-controlled reads at the genus or species level is shown in Fig. 1. Among the nine sequencing conditions at genus level, the lowest values of both dissimilarity index with the expected abundance of the mock community (0.22) and the percentage of “mis- or unidentified” (1.3%) were observed in a long-read combination of KAPA2G™, rRNA operon, and ncbi_202006 DB. At the species level, the same combination obtained the lowest values (0.23 of dissimilarity index and 6.2% of “mis- or unidentified”). The Illumina MiSeq platform targeting the V3-V4 region obtained 0.40 of dissimilarity index and 9.4% of “mis- or unidentified” at genus level and these values were higher than any long-read combination values obtained in this study. The values of the short-read method at species level (0.52 of dissimilarity index and 23.9% of “mis- or unidentified”) were inferior to the best long-read method (KAPA2G™, rRNA operon, and ncbi_202006 DB). In addition, numbers of taxon misidentified, i.e., assigned to genus or species other than the 15 bacteria in the mock bacterial community, from Illumina MiSeq (8 genera and 17 species, respectively) were higher than the average numbers of full-length 16S rRNA gene (4.8 and 14.8) and rRNA operon (3.5 and 5.3) (Fig. S1). Although short-read sequencing method is the most commonly used in the field of metagenomic data analysis in recent years^8^, we observed the rRNA operon amplicon sequencing was superior to the short-read sequencing in terms of dissimilarity index with the expected abundance of the mock community, percentage of “mis- or unidentified”, and numbers of taxon misidentified. Therefore, we focused on long-read sequencings in this study.

The beta diversities among the eight long-read sequencings were explained mainly by PCR target region (genus level, *R*^2^ = 0.51946, *P* = 0.0042; species level, *R*^2^ = 0.55341, *P* = 0.0036, PERMANOVA, Table 1). The PCR polymerase kits and reference databases had less effect on the taxonomic assignment than the PCR target regions, but still affected both the dissimilarity from the expected abundance of the mock community and the percentage “mis- or unidentified”. In total, the combination of KAPA2G, rRNA operon, and ncbi_202006 DB performed best at assigning the genus and particularly the species in terms of both dissimilarity and percentage “mis- or unidentified”. Therefore, we compared shotgun sequencing method and two amplicon sequencing methods in the following experiments: KAPA2G + rRNA operon + ncbi_202006 DB and KAPA2G + full-length 16S rRNA gene + ncbi_202006 DB.

**Table 1:** PERMANOVA of mock community.

Taxonomic level	Effect	Df	SS	MS	F.Model	R2	Pr(> F)
Genus level	PCR target region	1	0.20243	0.202433	8.3050	0.51946	0.0042
	PCR polymerase kit	1	0.05565	0.055651	2.2831	0.14281	0.0738
	Reference Database	1	0.03411	0.034112	1.3995	0.08754	0.2941
	Residuals	4	0.09750	0.024375		0.25019	
	Total	7	0.38970			1	
Species level	PCR target region	1	0.29283	0.292825	9.3314	0.55341	0.0036
	PCR polymerase kit	1	0.04329	0.043285	1.3794	0.08180	0.3081
	Reference Database	1	0.06750	0.067499	2.1510	0.12757	0.1667
	Residuals	4	0.12552	0.031381		0.23722	
	Total	7	0.52913			1	

Model includes PCR target region, PCR polymerase kit, and reference database.

*Df* degrees of freedom; *SS* sum of squares; *MS* mean squares; *R*^2^ coefficient of determination.

### Effect of inadequate database

To examine the influence of an inadequate database, we compared the mapping results by using ncbi_202006 DB and the incomplete database which did not contain *Bacillus subtilis* and *Lactobacillus delbrueckii*. The deleted species were not detected at all when used the incomplete database. There was a significant increase in the percentage of mis- or unidentified in both amplicon sequencings, while no significant change was observed in the percentage of bacteria other than the two deleted species (Fig. S2). This result shows the importance of using an enriched database that includes a wide range of bacterial species.

### Unlinked rRNA genes

Unlinked rRNA genes are widespread in natural environments such as soil but have not been detected in the human gut^17^. The presence or absence of unlinked rRNA genes in the gut of other animals has been unclear. Here, we found sequence reads of unlinked rRNA genes in all three equine fecal samples (Tables 2, S5). In particular, the feces from horse L were collected directly from the rectum, with no opportunity for contamination with unlinked rRNA genes from environments such as straw beds and soils. Therefore, we believe that the unlinked rRNA genes are naturally present in horse feces. All reads with unlinked rRNA genes found from the horses were mapped in KMA software^22^ to genus *Cellulosilyticum* or *Anaerocolumna* and were quality filtered by CCMetagen software^23^ to family *Lachnospiraceae*, order *Clostridiales*, or phylum *Firmicutes*, depending on their mapping qualities. Members of the *Lachnospiraceae* can ferment diverse plant polysaccharides to produce short-chain fatty acids^32^, and make up the largest single group in rumen microbes of cows^33^. No unlinked rRNA gene in three complete genomes of genus *Anaerocolumna* was found (Tables S6, S6_1–3). The complete genome of *Anaerocolumna aminovalerica*, which was mapped by a sequence read in this study, was not registered in the National Center for Biotechnology Information (NCBI) database as at the end of 2020, and the presence of unlinked rRNA genes in *Anaerocolumna* remains to be studied. On the other hand, we found that the genome of *Cellulosilyticum lentocellum* DSM 5427 has multiple rRNA copies with a combination of linked- and unlinked rRNA genes, being defined as “mixed type”^17,34^ (Tables S6, S6_4). We confirmed that *Cellulosilyticum* sp. WCF-2 (accession no. NZ_CP034675), which was isolated from cow feces, also has unlinked rRNA genes (Table S6_5). Therefore, we believe that the equine gut microbiota, unlike that in the human gut, contains unlinked rRNA genes, and that other herbivores—at least cows—also might have unlinked rRNA genes in the gut microbiota. The average length of the ITS regions in unlinked rRNA genes was almost 410,000 bp^17^, and could not be PCR-amplified before sequencing because of its long length. So rRNA operon amplicon sequencing might underestimate the occurrence of these strains with unlinked rRNA genes. We evaluated the degree of underestimation next.

**Table 2:** Detailed information of sequence reads with unlinked rRNA genes.

Horse	Sequence id	Read mapping in KMA software with ncbi 202006 DB	Taxonomic assignment in CCMetagen software
A	22eb24f6-ab8c-4935–9581-c65b2a9d1afa	642492|NC_015275.1 *Cellulosilyticum lentocellum* DSM 5427	*Clostridiales*
	9cfd48d6-1be8-4f1e-b72a-fef9e5495205	1527|NZ_OAOG01000091.1 *Anaerocolumna aminovalerica*	*Lachnospiraceae*
S	dd8fdda9-49f6-4d6a-9c09-ccee93bb6a59	642492|NC_015275.1 *C. lentocellum* DSM 5427	*Firmicutes*
L	15fc239f-2794-40e4-acaa-1a8e1d31cc07	1294025|NZ_BBCG01000153.1 *C. ruminicola* JCM 14822	*Lachnospiraceae*
	45df32f4-6390-487a-99d4-75ef1b78e773	642492|NC_015275.1 *C. lentocellum* DSM 5427	*Clostridiales*
	715b9279-6443-4a46-a258-2972c2d64712	1294025|NZ_BBCG01000153.1 *C. ruminicola* JCM 14822	*Lachnospiraceae*

### CCMetagen pipeline and Healthy fecal samples

**Figure 2:**
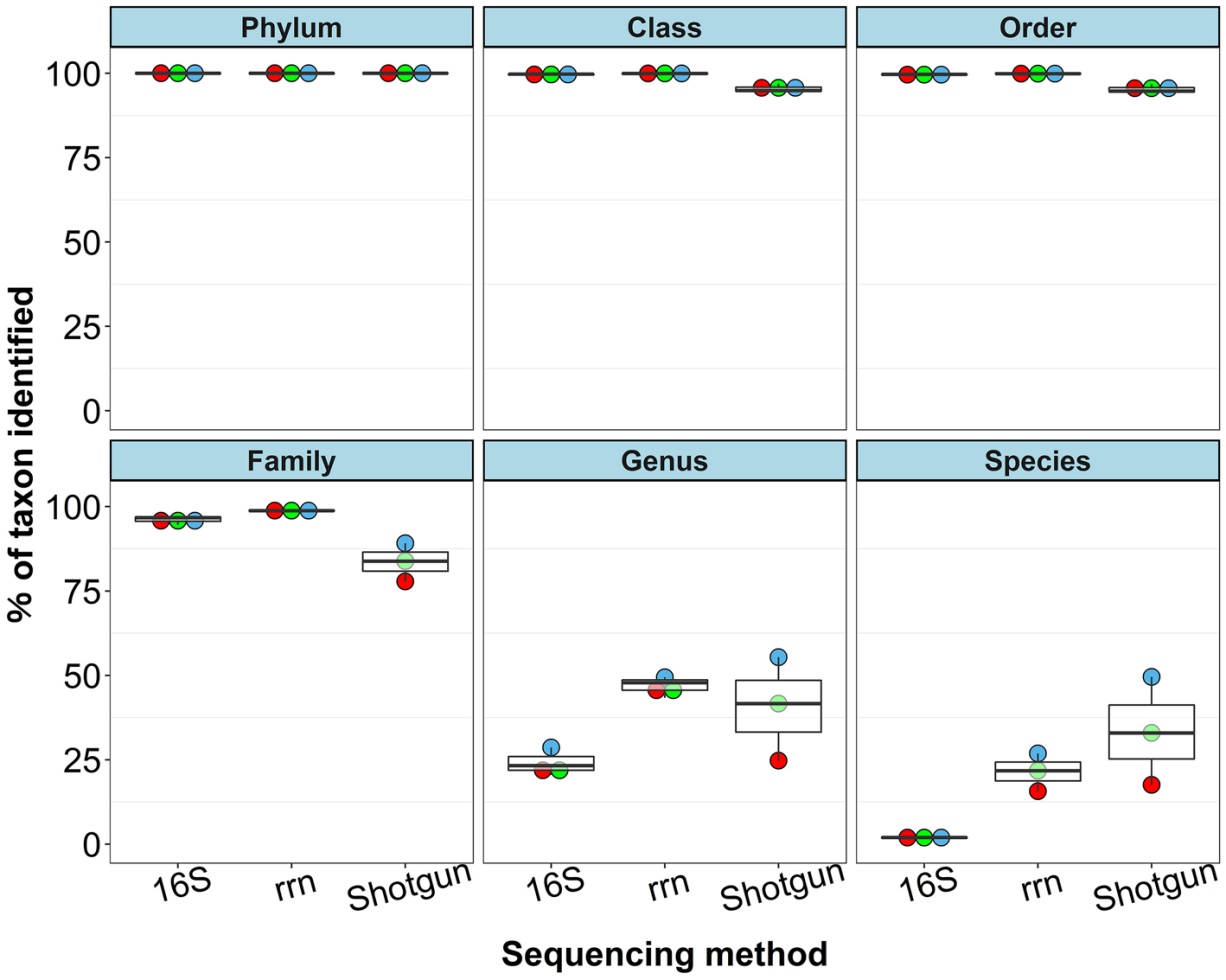
Rates of reads assigned to each taxon from phylum to species level. Each point shows each horse: red, horse A; blue, horse S; green, horse L. *16S* full-length 16S rRNA gene amplicon sequencing. *rrn* rRNA operon amplicon sequencing, *Shotgun* shotgun sequencing.

**Figure 3:**
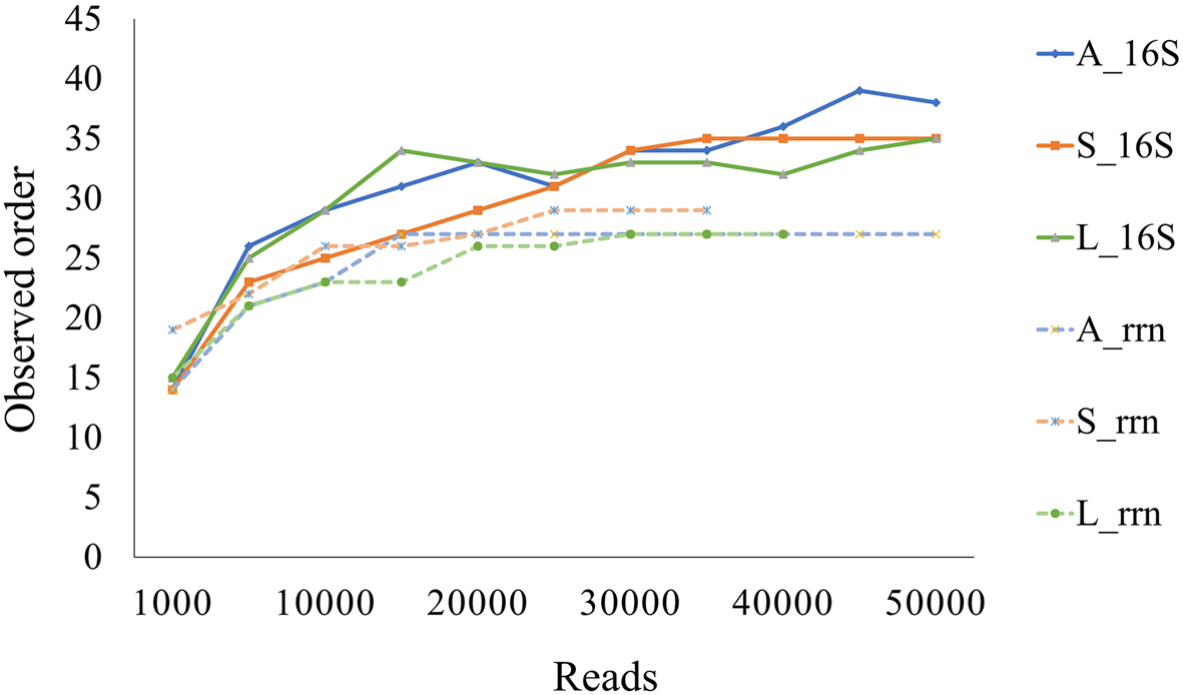
Numbers of observed orders against numbers of sequence reads. Three equine fecal samples (A, S, and L) were subjected to full-length 16S rRNA gene amplicon sequencing (16S) and rRNA operon amplicon sequencing (rrn).

Clustering of operational taxonomic units (OTUs) and amplicon sequence variant analysis are commonly conducted for 16S amplicon sequencing on the Illumina platform. These steps can reduce duplications and errors of representative sequences, resulting in a more reliable taxonomic assignment^35,36^. But there are no solid consensus methods for OTU clustering or amplicon sequence variant analysis to obtain accurate taxonomic assignment in nanopore sequencing, and the latter’s higher error rate could overestimate the bacterial diversity in samples^16,37^. To avoid overestimation, several methods have been used with nanopore data: e.g., reconstructing the consensus sequence^16^ or removing singleton reads or OTUs, or removing reads or OTUs whose percentages are less than arbitrary values such as 0.005%^12,38^. In addition, the taxonomic identification of metagenomic reads is generally more reliable at higher taxonomic levels (e.g., phylum or class) than at lower levels (e.g., genus or species), but it is not easy to decide which taxonomic level is reliable enough, because it varies by metagenomic read. Here, we used a CCMetagen pipeline to obtain accurate taxonomic assignments at appropriate taxonomic levels by conducting quality-filtering steps in CCMetagen software after the read mappings in KMA software. This pipeline reportedly outperforms current analytic methods (i.e., Kraken2, Centrifuge, and KrakenUniq) in making accurate taxonomic classifications^23^. Although comparisons with other assignment methods to apply to nanopore reads remain to be made, the CCMetagen pipeline revealed that the average percentages of taxonomic identification at phylum, class, and order levels in fecal samples were > 90% by all three sequencing sources (Fig. 2). In addition, the average percentages at the family level by the two amplicons were also high enough to use for downstream analysis: 96.1% by 16S rRNA sequencing and 98.8% by rRNA operon sequencing. The percentages of taxa identified dropped sharply at genus level to 24.1% by 16S rRNA sequencing and 46.9% by rRNA operon sequencing, while those for the mock community were still comparatively high at genus level (93.3% and 98.7%, respectively) (Figs. 1, 2). These results might be attributed to the compositional complexities of the fecal samples. In addition, as the results in the mock community using the incomplete database, the deficits of registered genome information in the public databases of relevance to the equine gut microbiota might also have affected these results. The results at the order level, which are the lowest among the taxa with a taxonomic identification rate of ≥ 90% in all sequencing methods, were used for downstream analysis. Depiction of the numbers of orders detected against the numbers of reads analyzed showed that the curve started to plateau at around 30,000 reads (Fig. 3). So we randomly subsampled 30,000 reads from each combination of analytical conditions and used them in further experiments to analyze the composition of the equine gut microbiota.

We first compared the three sequencing methods in terms of the unlinked rRNA genes. The percentages of genus *Cellulosilyticum* and *Anaerocolumna* are shown in Table S7. The percentages of *Cellulosilyticum* which was found to possess unlinked rRNA genes ranged from 0.53 to 1.54% in horse A, 0.31 to 1.00% in horse S, and 0.14 to 0.73% in horse L. The total percentages including genus *Anaerocolumna*, although further study is needed to confirm the presence of the unlinked rRNA genes in *Anaerocolumna,* ranged from 0.88 to 2.81% in horse A, 0.53 to 2.03% in horse S, and 0.26 to 1.22% in horse L, showing that the values differed slightly depending on the sequencing method. The average values of the three horses with or without *Anaerocolumna* by rRNA operon amplicon sequencing (0.33% and 0.55%, respectively) were roughly a fourth to a third of the percentages by the other two methods (0.99% and 2.02% by 16S rRNA amplicon sequencing, 1.02% and 1.42% by shotgun sequencing). These results showed that bacterial strains with unlinked rRNA genes could account for approximately 1–2% of the equine fecal microbiota and amplicon sequencing targeting the rRNA operon underestimated the percentages of bacterial species with unlinked rRNA genes.

**Figure 4:**
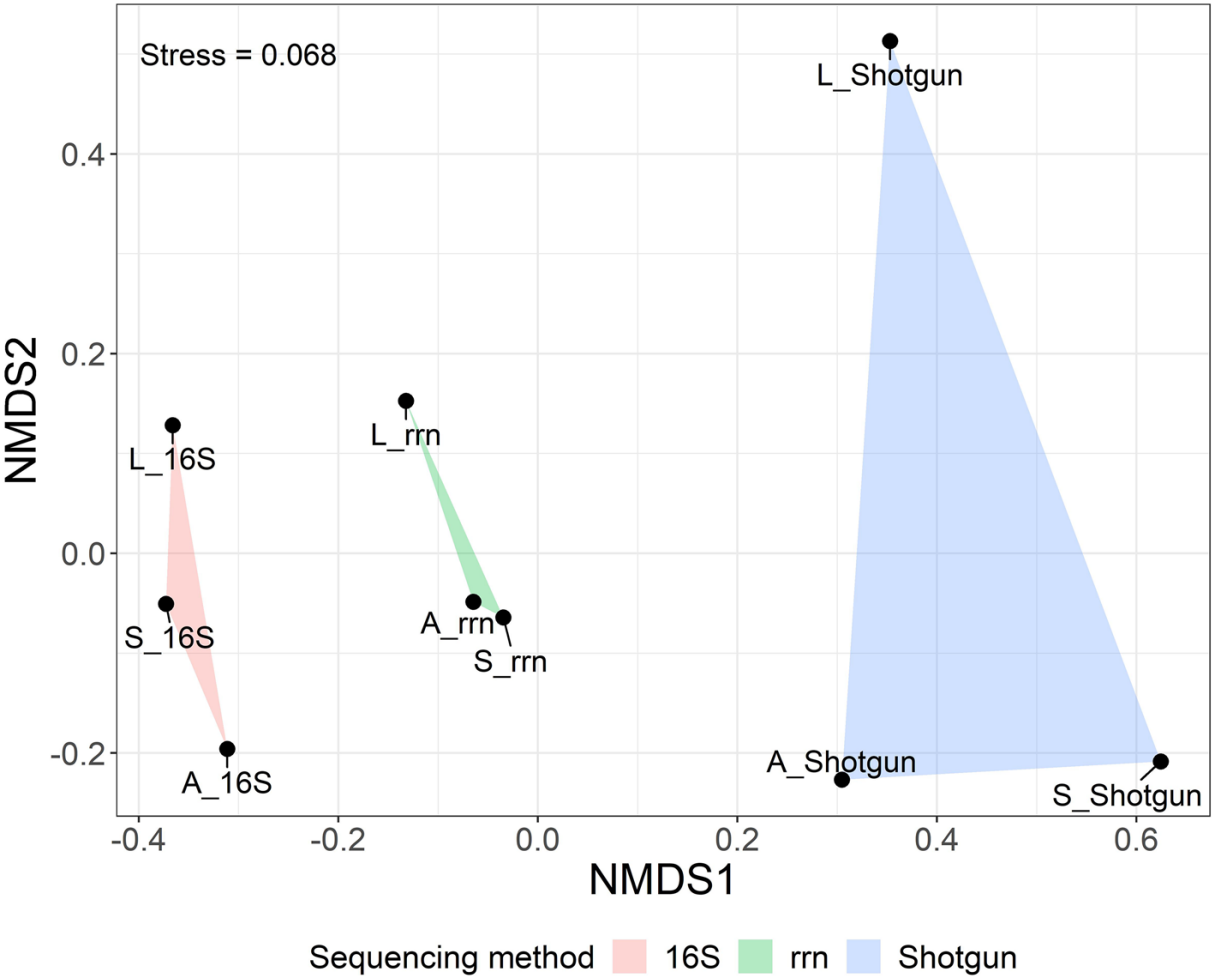
NMDS analysis of the microbial communities in equine fecal samples. Three equine fecal samples (A, S, and L) formed significant groups by sequencing method (PERMANOVA: *P* = 0.0037, *R*^2^ = 0.74328). 16S: full-length 16S rRNA gene amplicon sequencing. *rrn* rRNA operon amplicon sequencing, *Shotgun* shotgun sequencing.

**Figure 5:**
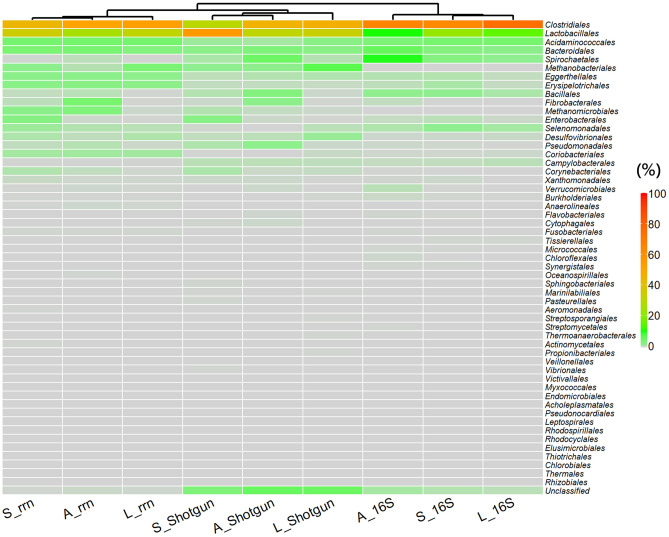
Heat map of each order assigned and hierarchical clustering of sample types. The hierarchical clustering shows that the samples from the amplicon sequencing with rRNA operon are grouped with those from shotgun sequencing. Sample types are shown as in Fig. 4.

To evaluate the influence of the underestimation of unlinked rRNA genes on the overall metagenomic results, we performed non-metric multidimensional scaling (NMDS) analysis. The taxonomic groups of the metagenomes generated from the same sequencing method were significantly similar (PERMANOVA: *P* = 0.0037, *R*^2^ = 0.74328), but the individual horse did not have a significant effect (PERMANOVA: *P* = 0.6553, *R*^2^ = 0.07468). The analysis showed clear differences among the three sequencing methods (Fig. 4). Despite the influence of the unlinked rRNA genes, the plots of the rRNA operon were distributed between those of the other methods, not generating an obvious independent group as compared to the other sequencings. In addition, clustering analysis shown by heat map grouped the compositions of the rRNA operon with those from the shotgun sequencing, showing that rRNA operon amplicon sequencing did not make an independent group by itself (Fig. 5). Overall, we found that amplicon sequencing targeting the rRNA operon could underestimate the percentage of bacterial strains containing unlinked rRNA genes by a fourth to a third when applied to equine fecal samples, but the effects on the overall metagenomic results were limited, and the operon targeted sequencing could generate similar metagenomic results to those by shotgun sequencing, which has no PCR bias.

**Figure 6:**
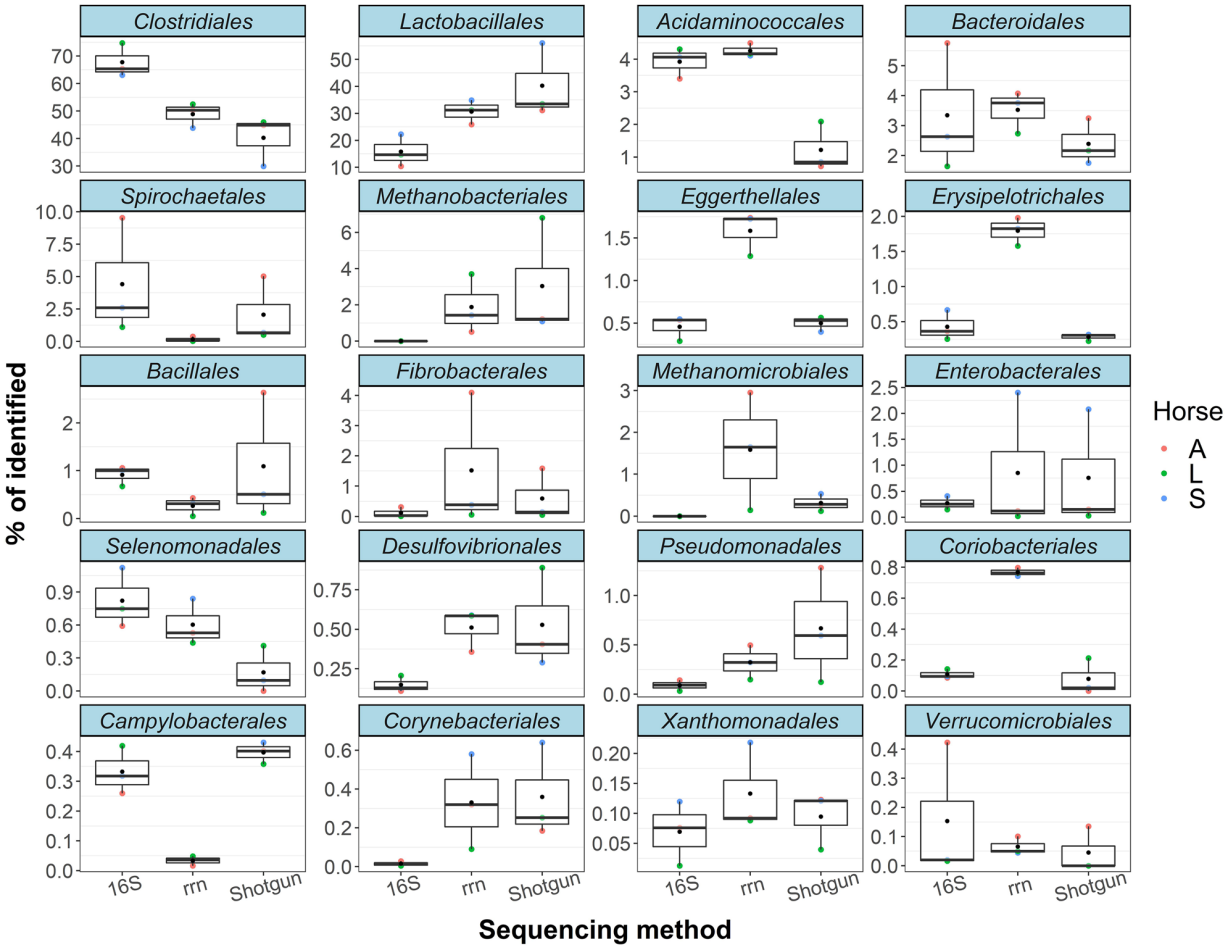
Boxplots of the top 20 orders with the highest average percentages. Sequencing methods are shown as in Fig. 2. Average values of the three horses were shown as black dot.

We further explored differences between the three sequencing methods. Orders *Clostridiales* and *Lactobacillales* were dominant in all fecal samples by all methods (Figs. 5, 6). The averages of Orders *Clostridiales* and *Lactobacillales* were 67.7% and 15.8% in 16S rRNA gene sequencing, 48.9% and 30.7% in rRNA operon sequencing, and 40.3% and 40.2% in shotgun sequencing, respectively. These results are compatible with previous reports that the *Firmicutes*, which encompass the *Clostridiales* and the *Lactobacillales*, was the most dominant taxon in fecal samples from healthy Thoroughbreds^39,40^. Other taxa were much less predominant, but several taxa from each sequencing method had considerably lower abundances than those from the other sequencing methods: e.g., *Desulfovibrionales* and *Corynebacteriales* in 16S rRNA sequencing, *Spirochaetales* and *Campylobacterales* in rRNA operon sequencing, and *Acidaminococcales* in shotgun sequencing (Fig. 6). Several factors are known to affect taxonomic assignment, including primer sets, PCR conditions, and reference databases^18,41–43^. We used the same database, ncbi_202006 DB, in all the three sequencing methods, and attribute the differences described above mainly to primer mismatch or to PCR conditions such as annealing temperature. The most notable and interesting differences among the three sequencing methods were found in the archaea, in the orders *Methanobacteriales* and *Methanomicrobiales*. While 16S rRNA gene sequencing with the 27F + 1492R primer set could not find any archaeal genomes, both rRNA operon sequencing and shotgun sequencing could detect archaea, at mean values of 3.5% (1.9% of *Methanobacteriales* and 1.6% of *Methanomicrobiales*) and 3.3% (3.0% and 0.3%), respectively (Fig. 6). These archaea produce methane from H_2_ and CO_2_ and might boost the carbohydrate-degrading activity of cellulolytic bacteria^4,6^; therefore, methanogenic archaea should be more intensively focused on to unveil the mechanisms and functional interactions among microbes needed for successful degradation of nutrients. The diversity and importance of the archaea in various environments remain poorly understood^44^; one reason is that few primer sets have been validated^45^. The primer set used for the rRNA operon amplicon sequencing in this study—A519F + U2428R—covers a large proportion of the known bacterial and archaeal rRNA genes^15^, and the ratio of archaea is comparable to the results obtained by shotgun sequencing. Although the rRNA operon amplicon sequencing with the primer set of A519F + U2428R could detect some kind of archaeal genomes such as *Methanobacteriales* and *Methanomicrobiales*, the lack of archaeal genomes in the mock community could be a major limitation in this study, and further validation is needed for archaea.

To validate the reproducibility of the rRNA operon amplicon sequencing and analysis pipeline after DNA extraction, two additional sequencings per horse sample were conducted. The results showed that the characteristic differences among the fecal samples, such as *Fibrobacterales*, *Methanomicrobiales*, *Enterobacterates*, were reproduced, albeit with some variation among the three examinations. (Fig. S3). Furthermore, we were able to confirm the reproducibility by NMDS analysis (Fig. S4) and found that there was sufficient reproducibility in the steps after DNA extraction.

## Conclusions

We compared long amplicon sequencing targeting the rRNA operon with the A519F + U2428R primer set with short-read sequencing method targeting V3-V4 region and other long-read sequencing methods, i.e., amplicon sequencing targeting the full-length 16S rRNA gene with the 27F + 1492R set and shotgun sequencing. Even though full-length 16S rRNA gene amplicon sequencing has low taxonomic resolution at the genus and especially species levels and cannot evaluate archaeal genomes, the 16S rRNA gene is still a promising target for metagenomic analysis using long amplicons because of its robustness among bacterial communities and the availability of comprehensive databases. rRNA operon amplicon sequencing combined with the KAPA2G™ Robust HotStart Ready Mix PCR Kit and the ncbi_202006 DB, a newly designed reference database, performed best at assigning the genus and particularly the species by using a bacterial mock community. Besides, the rRNA operon amplicon sequencing had fewer numbers of taxon misidentified compared to the short-read and full-length 16S rRNA gene sequencings. The rRNA operon amplicon sequencing detected some kind of archaeal genomes such as *Methanobacteriales* and *Methanomicrobiales* in equine feces, which might be an advantage of the A519F + U2428R primer set. In addition, the rRNA operon amplicon sequencing could offer confident bacterial taxonomic assignments even at lower ranks, so researchers can capture fecal microbiota in more detail in horses.

## Supplementary Information

Supplementary Information 1.

Supplementary Information 2.

## Data Availability

All nanopore sequencing data generated in this study are available under the BioProject accession number PRJDB10841, with BioSamples accession numbers SAMD00260421 (mock community), SAMD00260422 (horse A), SAMD00260423 (horse S), and SAMD00260424 (horse L).
