# Supplementary material for: Establishment and assessment of an amplicon sequencing method targeting the 16S-ITS-23S rRNA operon for analysis of the equine gut microbiome
Source: Sci Rep. 2021 Jun 4;11:11884. doi: 10.1038/s41598-021-91425-7 (PMC8178347; doi:10.1038/s41598-021-91425-7)
Supplement: Supplementary file 1 — Supplementary Information 1. [file 41598_2021_91425_MOESM1_ESM.pdf]

Establishment and assessment of an amplicon sequencing method targeting the 16S-ITS-23S rRNA operon for analysis of the equine gut microbiome

Yuta KINOSHITA[1,*], Hidekazu NIWA[1], Eri UCHIDA-FUJII[1], Toshio NUKADA[1]

[1]Microbiology Division, Equine Research Institute, Japan Racing Association, 1400-4 Shiba, Shimotsuke, Tochigi 329-0412, Japan

[*] Correspondence to: Yuta Kinoshita

Microbiology Division, Equine Research Institute, Japan Racing Association, 1400-4 Shiba, Shimotsuke, Tochigi 329-0412, Japan.

kinoshita@equinst.go.jp

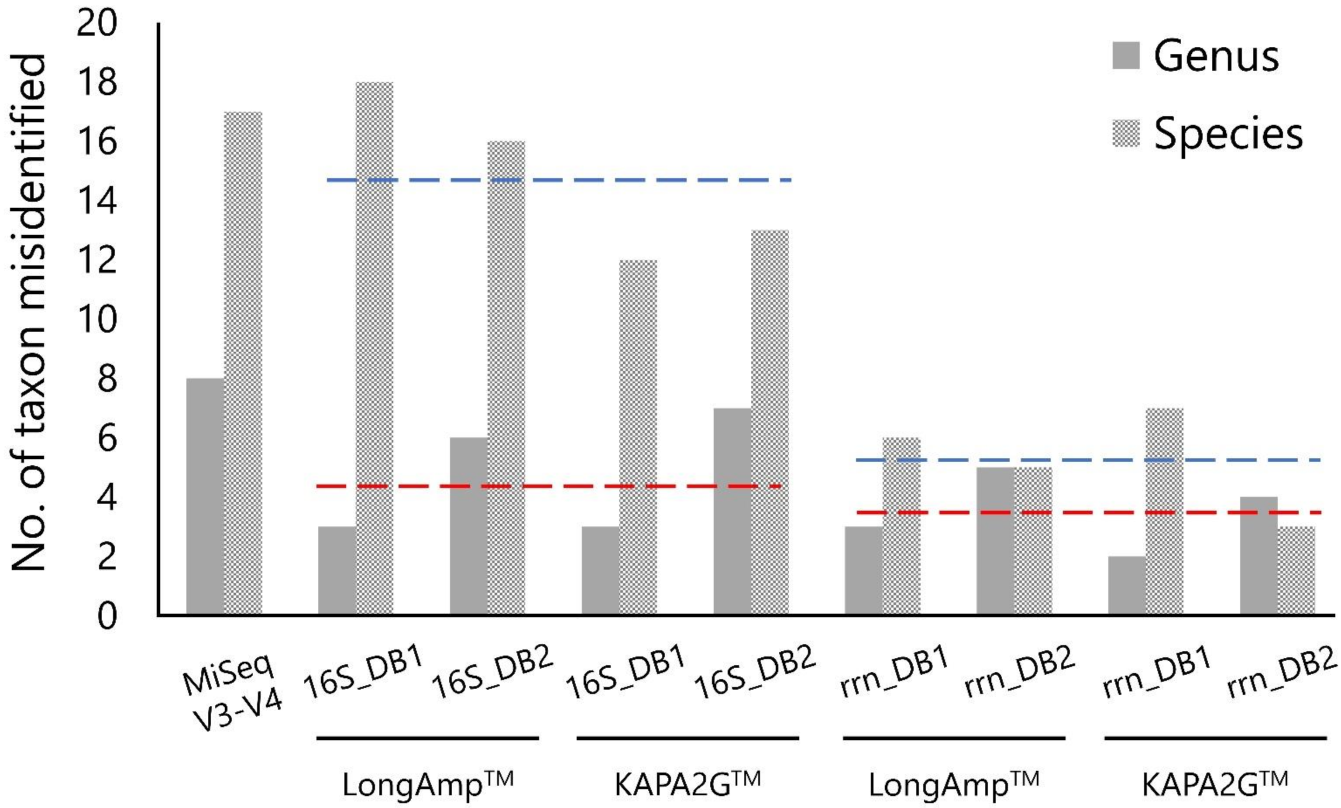

Figure S1: Numbers of taxon misidentified at genus and species level. Red and blue dash lines indicate the average number of 16S rRNA gene amplicon sequencing and rRNA operon amplicon sequencing at species and genus levels, respectively. 16S: full-length 16S rRNA gene amplicon sequencing. rrn: rRNA operon amplicon sequencing. DB1: rrn DB[24]. DB2: ncbi_202006 DB.

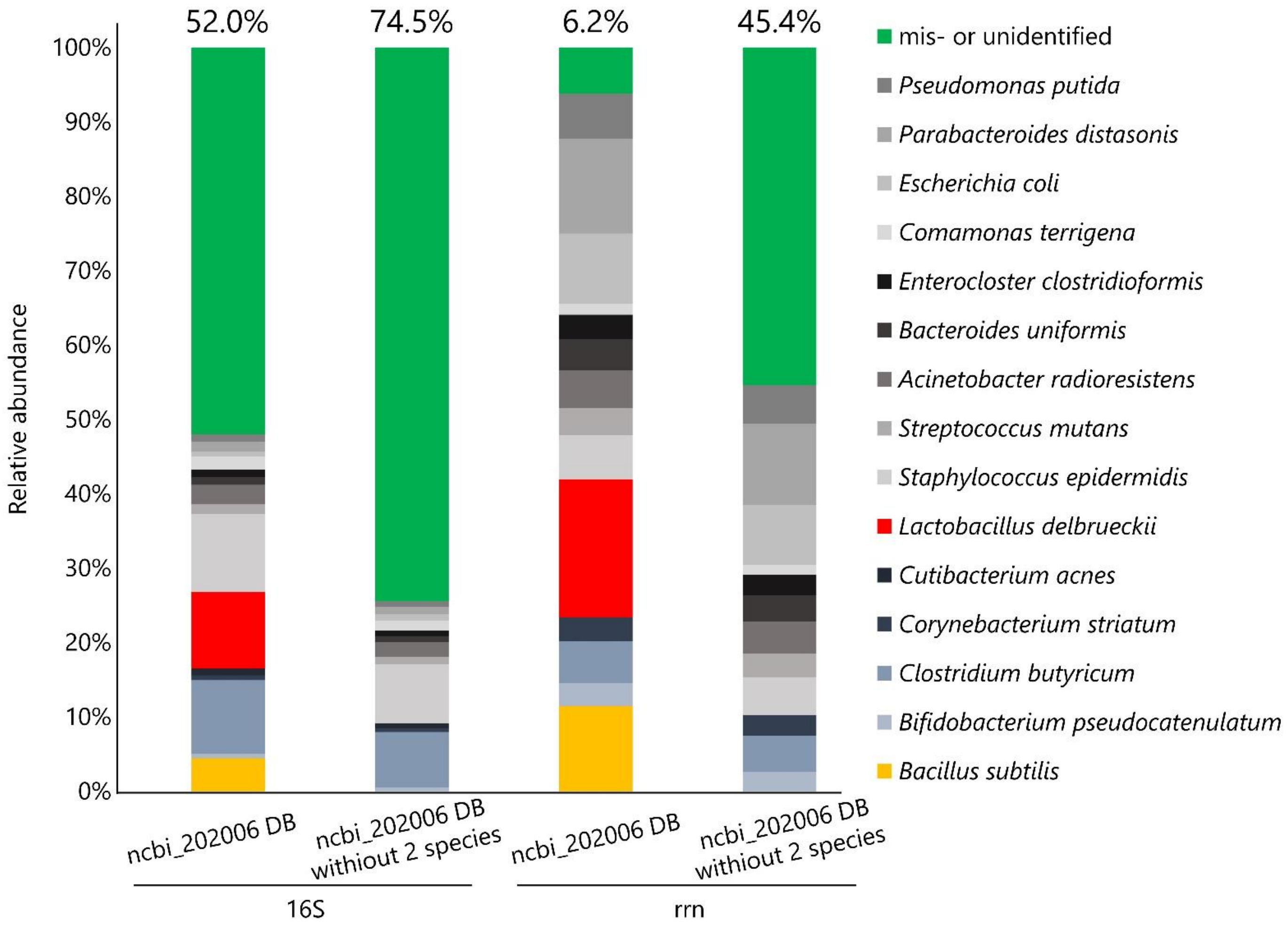

Figure S2: Influence of an inadequate database on read mapping. Relative abundance of mock community using two databases are shown: 1) ncbi_202006_DB and 2) ncbi_202006_DB without *Bacillus subtilis* and *Lactobacillus delbrueckii*. The percentages of mis- or unidentified sequences are shown above each bar graph. 16S: full-length 16S rRNA gene amplicon sequencing. rrn: rRNA operon amplicon sequencing.

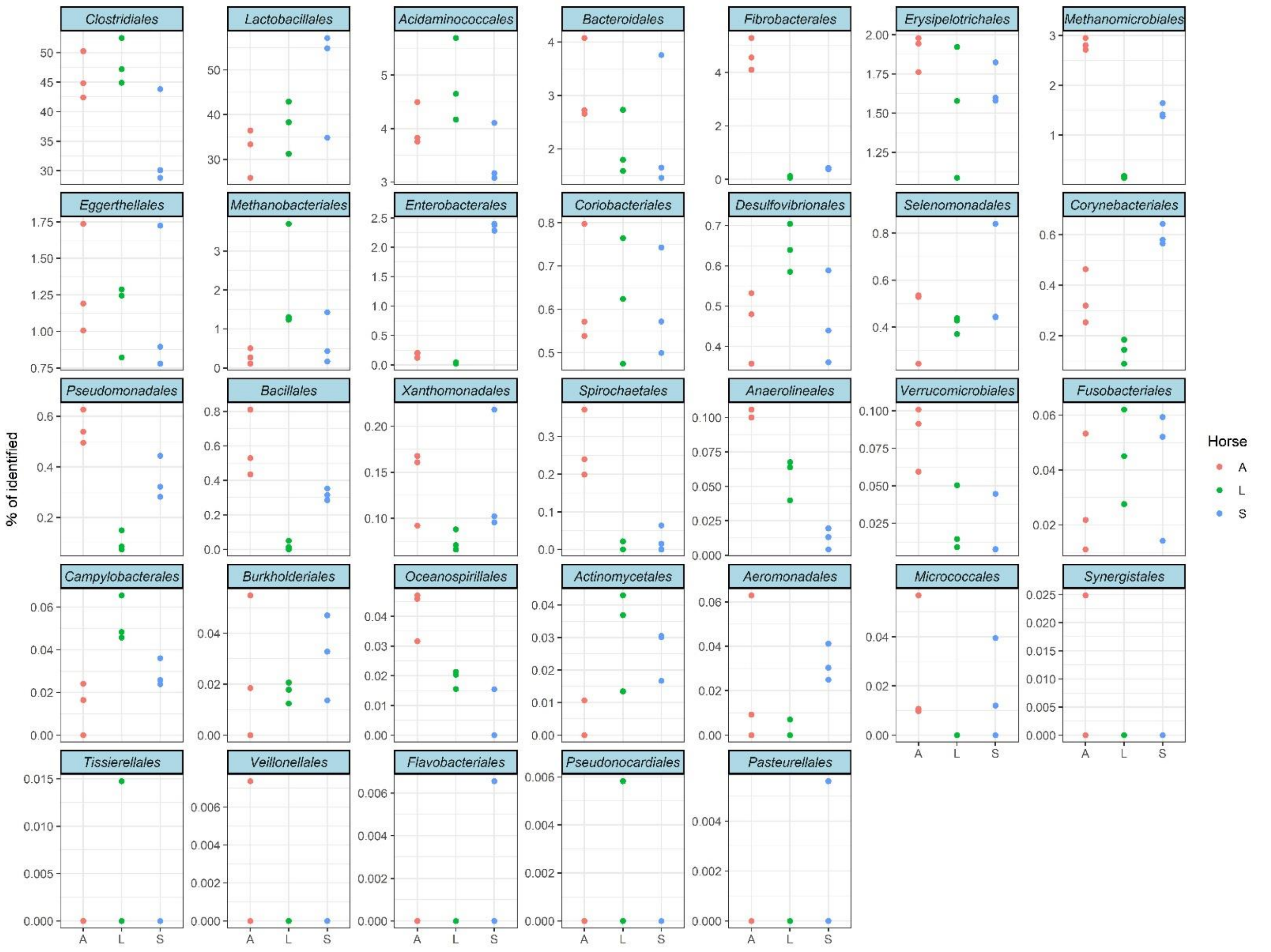

Figure S3: Boxplots of a percentage of identified order using rRNA operon amplicon sequencing in equine fecal samples. Each point shows each horse: red, horse A; green, horse L; blue, horse S.

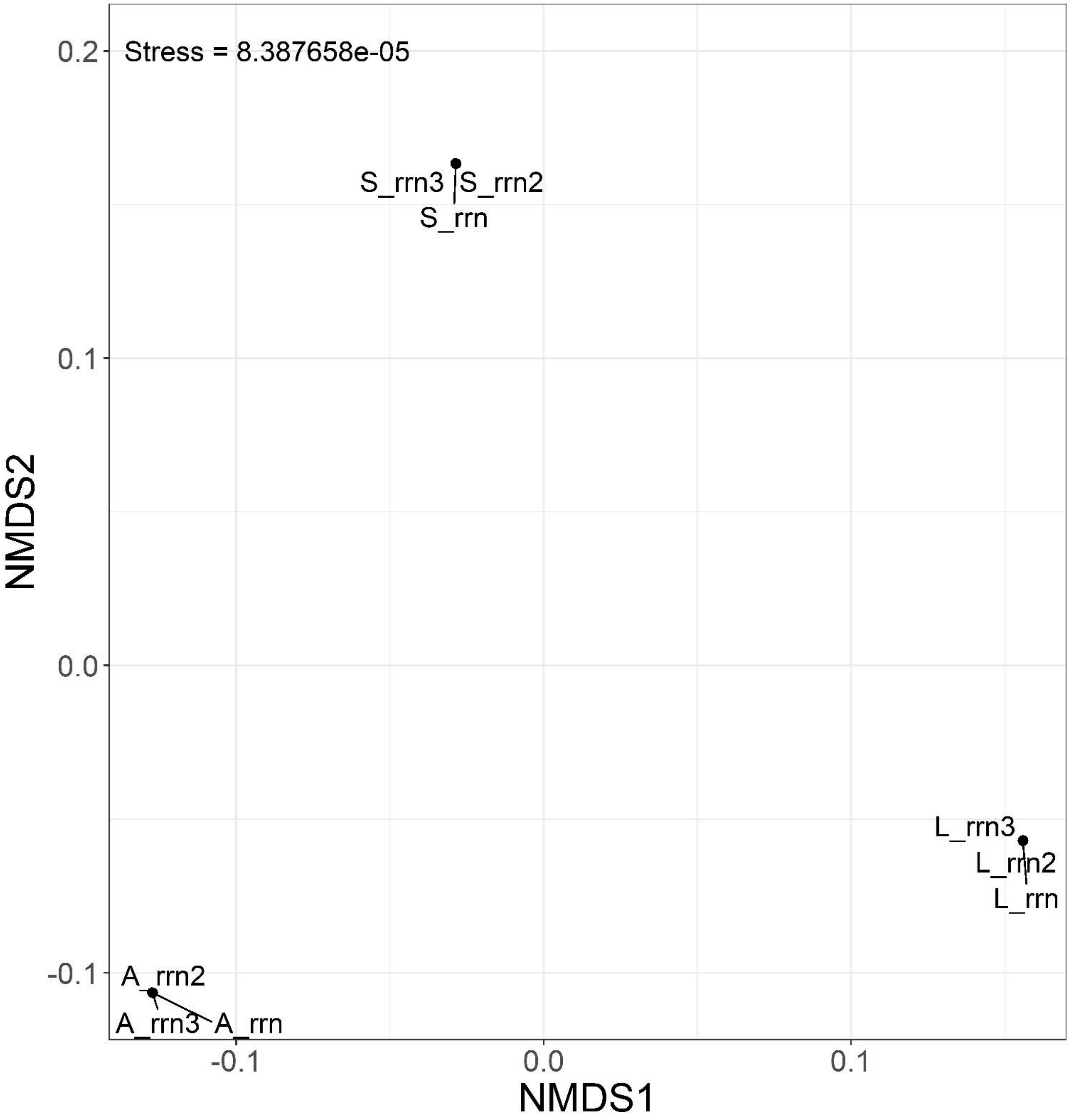

Figure S4: NMDS analysis of the equine fecal microbiota using rRNA operon amplicon sequencing. Three amplicon sequencings were performed per horse (A, S, and L). rrn: rRNA operon amplicon sequencing.
